# The Molecular Aspect of Antitumor Effects of Protease Inhibitor Nafamostat Mesylate and Its Role in Potential Clinical Applications

**DOI:** 10.3389/fonc.2019.00852

**Published:** 2019-09-03

**Authors:** Xi Chen, Zhijie Xu, Shuangshuang Zeng, Xiang Wang, Wanli Liu, Long Qian, Jie Wei, Xue Yang, Qiuying Shen, Zhicheng Gong, Yuanliang Yan

**Affiliations:** ^1^Department of Pharmacy, Xiangya Hospital, Central South University, Changsha, China; ^2^National Clinical Research Center for Geriatric Disorders, Xiangya Hospital, Central South University, Changsha, China; ^3^Department of Pathology, Xiangya Hospital, Central South University, Changsha, China

**Keywords:** nafamostat mesylate, antitumor activities, signaling pathways, efficacy, toxicity

## Abstract

Nafamostat mesylate (NM), a synthetic serine protease inhibitor first placed on the market by Japan Tobacco in 1986, has been approved to treat inflammatory-related diseases, such as pancreatitis. Recently, an increasing number of studies have highlighted the promising effects of NM in inhibiting cancer progression. Alone or in combination treatments, studies have shown that NM attenuates various malignant tumors, including pancreatic, colorectal, gastric, gallbladder, and hepatocellular cancers. In this review, based on several activating pathways, including the canonical Nuclear factor-κB (NF-κB) signaling pathway, tumor necrosis factor receptor-1 (TNFR1) signaling pathway, and tumorigenesis-related tryptase secreted by mast cells, we summarize the anticancer properties of NM in existing studies both *in vitro* and *in vivo*. In addition, the efficacy and side effects of NM in cancer patients are summarized in detail. To further clarify NM's antitumor activities, clinical trials devoted to validating the clinical applications and underlying mechanisms are needed in the future.

## Introduction

Cancer is a worldwide public health problem and has been the major cause of death in recent years (1). Current effective modalities for curing cancers include radiation, surgery, and drugs. Among these therapeutic approaches, chemotherapy is indispensable for tumor therapy and has prolonged the lives of many patients over the past decades. However, treatment failure and side effects are common in chemotherapy, which adversely influence both patient survival and quality of life. Thus, there is a huge demand for powerful chemotherapeutic agents that reduce side effects and provide increased survival benefits for cancer patients. Recently, increasing studies have indicated that protease inhibitors suppress tumor growth and progression (2, 3). In particular, several protease inhibitors, such as bortezomib, carfilzomib, and ixazomib, have been approved by the United States Food and Drug Administration (FDA) for clinical use in the treatment of multiple myeloma (4). All of these findings suggest that protease inhibitors have broad prospects for development as anticancer agents.

Nafamostat mesylate (NM), also known as FUT-175 and 6′-amidino-2-naphthyl-4 -guanidinobenzoate dihydrochloride, is a broad-spectrum serine protease inhibitor synthesized by Fujii et al. (5). Five years later, a company called Japan Tobacco brought it to market. NM is usually used to treat pancreatitis, disseminated intravascular coagulation (DIC), and systemic inflammatory response syndrome via suppression of thrombin, plasmin, kallikrein, trypsin, and Cl esterase in the complement system, as well as factors VIIa, Xa, and XIIa in the coagulation cascade (6–8). A growing contingency of researchers have focused on the potential anticancer effects of NM. As early as 1992, one study confirmed that NM inhibits liver metastasis at concentrations of 10^−6^–10^−7^ M in colon adenocarcinoma, which first uncovered its putative effects in cancer therapy. Subsequently, NM has been proven to downregulate the expression of both matrix metalloproteinase−2 (MMP-2) and−9, vascular endothelial growth factor (VEGF) and transforming growth factor beta 1 (TGF-β1), secreted by tumor cells in head and neck squamous cell carcinoma, and NM inhibits angiogenesis and tumor invasion (9). Furthermore, Mander et al. reported the NM significantly inhibits proliferation, migration, and invasion in triple-negative breast cancer, which was identified both *in vitro* and *in vivo* (10). Moreover, NM reverses immune resistance induced by interferon-gamma (IFN-ɤ) as a method of increasing programmed cell death ligand-1 (PD-L1) expression in lung and pancreatic cancer (11). Currently, exploration of the antitumor effects of NM are in full swing.

In this report, we concentrate on existing evidence regarding the antitumor activity of NM and discuss the potential mechanisms for NM targeting in cancer. In addition, to evaluate the possibility of NM use in future clinical applications, the effectiveness and adverse effects of NM are also discussed.

## Mechanism of NM Anticancer Effects

**Figure 1:**
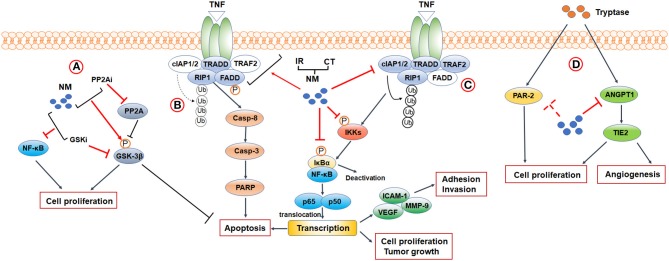
Mechanisms and biological functions of NM in cancer research. **(A)** PPAR2-GSK3β signaling. **(B,C)** A crosstalk between NF-κB signaling and apoptosis-related signaling. When TRAF2 is absent (indicated by blank color), c-IAP1/2 is no longer recruited, and RIP1 is not ubiquitinated (indicated by broken arrows). Non-ubiquitinated RIP1 induces caspase-8 dependent apoptosis. Activation of TNFR1 leads to the recruitment of TRADD, TRAF2, c-IAP1/2, and RIP1 to the TNFR1 complex. cIAP1/2 modifies RIP1 with polyubiquitin chains, leading to the activation of canonical NF-κB signaling. **(D)** Tryptase-mediated PAR-2 and ANGPT1/TIE2 signaling. CT, Chemotherapy; IR, Ionizing Radiation 3; PP2Ai, PP2A inhibitor; GSKi, GSK inhibitor.

So far, multiple studies have uncovered the potent anticancer abilities of NM. It is evident that NM inhibits cancer cells proliferation, adhesion and invasion, and suppresses tumor growth in animal models. Furthermore, NM initiates apoptosis both *in vitro* and *in vivo* (12–17). In addition, NM has the capacity to provide improvements in sensitivity of the tumor to conventional clinical treatments (15–25). Furthermore, as a synthetic serine protease inhibitor, interest is growing in the use of NM against tumor progression induced by MC-derived tryptase. Tumor cell proliferation and angiogenesis stimulated by tryptase was reversed by NM (26, 27). Herein, to investigate how NM exerts these anticancer effects, we discuss the mechanism for NM targeting (Figure 1).

### NF-κB Signaling

Nuclear factor-κB (NF-κB) is an inducible transcription factor comprising 5 family members, designated as NF-κB1/p50, NF-κB2/p52, RELA/p65, RELB, and c-REL, which bind to consensus DNA sequences at promoter regions as heterodimers and homodimers to activate target genes (28). So far, two major signaling pathways are considered to mediate NF-κB activation: the canonical and non-canonical NF-κB signaling pathways. In the canonical pathway, NF-κb activation occurs via degradation of the inhibitor of κB (IκB) family, consisting of characteristic members IκBα and several structurally related proteins, to which cytoplasmic NF-κB binds in a resting state. After stimulation by some stress or infection factor, the IκB kinase (IKK) complex, composed of catalytic (IKKα and IKKβ) and regulatory (IKKγ) subunits, is activated, subsequently leading to phosphorylation of IκBs. Phosphorylated IκBs further undergo ubiquitylation and proteasome-mediated degradation, promoting release and translocation of NF-κB dimers to regulate gene transcription (29). The entire canonical pathway induces activation of NF-κB heterodimers, including p50 and p65 or p50 and c-Rel, displaying rapid and transient characteristics. In contrast, activation of the non-canonical pathway generates p53 and RELB at a slow and persistent speed with involvement of only IKKα (30). Based on these two pathways, NF-κB activation mediates a wide variety of human disease, particularly cancers.

NF-κB is frequently hyperactivated in several cancers, and its subunits have crucial roles in tumor proliferation, migration and resistance to radiotherapy and chemotherapy. Although the canonical NF-κB signaling pathway has been extensively studied in all kinds of cancers, there still new breakthroughs occur in this field. For instance, in breast and lung cancer, inflammatory cytokines, such as IFN-α and tumor necrosis factor (TNF), activate signal transducer and activator of transcription 1 (STAT1) and NF-κB/p65 in brain metastatic cells in response to stimulation by carcinoma–astrocyte gap junctions composed of protocadherin 7 and connexin 43, leading to tumor growth and chemoresistance (31). The relationship between the upstream and downstream inflammatory cytokines and NF-κB has been intensively studied, and this research revealed a novel role of NF-κB in carcinoma–astrocyte interaction models. Grinberg-Bleyer et al. reported that only deficiency of c-Rel in NF-κB subunits could suppress the generation and maintenance of activated regulatory T cells for impeding immune tolerance. Mice lacking c-REL experienced delayed melanoma growth and enhanced antiprogrammed cell death-1 (PD-1) immunotherapy responses (32). Since p65 containing NF-κB complexes are primarily responsible for cellular activation responses, targeting only c-Rel may prevent undesirable side effects. In addition, investigations have focused on the interplay between malignancy and the non-canonical pathway in recent years. In human glioblastoma cells, transcription of the C250T mutant telomerase (TERT) promoter was reactivated by p52 cooperating with the E-twenty-six family of transcription factors, promoting further regulation of TERT transcription and tumorigenicity (33). Accordingly, targeting critical NF-κB subunits or dysfunction of upstream pathways leading to NF-κB activation may afford cancer patients considerable treatment prospects.

Recently, NM has been shown to potently inhibit canonical NF-κB pathways and further suppress the aggressive behavior of various cancer, such as pancreatic cancer (12–14), colorectal (15), gastric (16), and hepatocellular carcinoma (17). In the study of Uwagawa et al. NM was found to inhibit IκBα phosphorylation and NF-κB DNA-binding activity in a dose-dependent and time-dependent manner in pancreatic cancer cells (12). Another study showed that the activity of intercellular adhesion molecule-1 (ICAM-1), VEGF, and MMP-9, the downstream transcription target genes of NF-κB (34–36), were stifled owing to NM-induced suppression of NF-κB activity, inhibiting pancreatic cancer cell adhesion, anoikis, and invasion (13). Moreover, NM could significantly decreased NF-κB activation which reducing tumor growth, neovascularization and prolonged survival by apoptosis modulation in pancreatic *in vivo* models (13, 14). Researches over the past decade have demonstrated that NM inhibited proliferation and induced apoptosis by IκBα/NF-κB/p65 nuclear translocation and caspase families regulation in colorectal, gastric, and hepatocellular carcinoma cells (15–17). In *in vivo* model of colorectal, hepatocellular and gastric cancers, NM have confirmed effective antineoplastic properties with reduced diameters, volumes, and weights of tumors as a single agent (15–17).

Furthermore, aberrant activation of NF-κB signaling has been proven as one potential mechanism underlying chemotherapy and radiotherapy failure. Intriguingly, a function of NM for enhancing the sensitivity of chemotherapy and radiotherapy though inhibition of NF-κB activation was reported in pancreatic, gastric, gallbladder and hepatocellular cancer (Tables 1, 2). Previous researches have reported that the combination of NM and chemotherapeutics agents, such as oxaliplatin, gemcitabine, paclitaxel, and nab-paclitaxel could increase sensitivity of single-agent and combination chemotherapy in pancreatic cancer. The chemo-sensitization abilities of NM were mainly reported by downregulation of IκBα phosphorylation and generating synergistic cytotoxicity, which inhibited NF-κB activation. Previous researches have reported that the combination of NM and chemotherapeutics agents, such as oxaliplatin, gemcitabine, paclitaxel, and nab-paclitaxel could increase sensitivity of single-agent and combination chemotherapy in pancreatic cancer. The chemo-sensitization abilities of NM were mainly reported by downregulation of IκBα phosphorylation and generating synergistic cytotoxicity, which inhibited NF-κB activation (18–22). *In vivo* studies have also demonstrated that NM sensitizes cells to the chemotherapy and further inhibits pancreatic tumor growth (18–22). Similar studies have revealed that NM inhibited NF-κB activity in hepatocellular carcinoma, gallbladder and gastric cancers both *in vitro* and *in vivo*. Specifically, after treatment of NM combined with gemcitabine in gallbladder cancer (25), with paclitaxel in gastric cancer (16) or with TNF-α in hepatocellular carcinoma (17), depression of NF-κB activation was stimulated. Another study also confirmed that the combination of NM sensitized oxaliplatin-induced NF-κB/p65 activation via suppressing phosphorylated IκBα and IKKα/β in colorectal cancer, resulting in reduced cell proliferation, increased apoptosis *in vitro* and decreased tumor growth *in vivo* (15). NM was also shown to act as a radiosensitizer via inhibition of NF-κB in colorectal (24) and pancreatic cancer (23). Of note, except for dysfunction of NF-κB, dysregulation of Mdm2 results in increasing p53 expression in response to NM treatment combined with 5 Gy ionizing radiation (IR) in pancreatic cancer (23). A number of studies indicate that both NF-κB and p53 are activated in response to DNA damage by ionizing radiation and modulate each other's activities (37).

**Table 1:** The anti-tumor activities of combination of NM and chemotherapy/radiotherapy *in vitro*.

**Tumor**	**Combined therapeutics**	**Cell lines**	**Targeting signaling factors**	**Biological functions**	**Drug concentration/dosage of IR**	**References**
Pancreatic cancer	Oxaliplatin	Panc-1 cells	p65 and phosphorylated IκBα; Cleaved caspase-8/ PARP; c-IAP1/2	Inhibition of cell proliferation, induction of apoptosis	Oxaliplatin (20 μM) and NM (160 μg/ml)	(18)
	Gemcitabine	Panc-1 cells	p65	Induction of apoptosis	Gemcitabine (0.01 μM) and NM (80 μg/ml)	(19)
	Gemcitabine and TNF-a	MIAPaCa-2/AsPC-1 cells	p50, p65, and phosphorylated IκBα; TNF-α-TNFR1; Cleaved caspase-8/caspase-3/PARP; c-IAP1/2;	Inhibition of cell proliferation Induction of apoptosis and G1 arrest	TNF-α (10 ng/mL), gemcitabine (1 μM), and NM (80 μg/mL)	(20)
	Gemcitabine (GEM) plus nab-paclitaxel (nPTX)	Panc-1, MIA PaCa-2/AsPC-1 cells	p65; Cleaved caspase-8/ caspase-3;	Inhibition of cell proliferation Induction of apoptosis	GEM (1, 0.494, and 23.9 μM, respectively), nPTX (0.5, 0.683, and 4.9 mM, respectively) and NM (80 μg/ml)	(21)
	Paclitaxel	AsPc-1 cells	p65 and phosphorylated IκBα; Cleaved caspase-8/caspase-3;	Induction of apoptosis andG2/M arrest	Paclitaxel (10 μM) and NM (80 μg/mL)	(22)
	IR	Panc-1/MIA PaCa-2 cells	p65; cleaved caspase-8/caspase-3; Mdm2/p53/ p21^Waf1/Cip1^;	Inhibition of cell proliferation Induction of apoptosis and G2/M arrest	6Gy (2, 6, 10Gy for cell proliferation assay) and NM (80 μg/mL)	(23)
Colorectal cancer	Oxaliplatin	HCT 116/SW 1116 cells	p65, phosphorylated IκBα and phosphorylated IKKα/β; Cleaved caspase-9/caspase-3/PARP; phosphorylated Erk;	Inhibition of cell proliferation Induction of apoptosis	Oxaliplatin (25 μM) and NM (100 μM)	(15)
	IR	SW620/ DLD-1 cells	p65; Cleaved caspase-8/caspase-3/caspase-9/PARP; MMP-2 and MMP-9;	Inhibition of cell proliferation, migration and invasion Induction of apoptosis	5 Gy and NM (80 μg/mL)	(24)
gastric Cancer	Paclitaxel	MKN-45 cells	p65 and phosphorylated IκBα; Cleaved caspase-8/caspase-3/ PARP;	Inhibition of cell proliferation Induction of apoptosis and G1 arrest	Paclitaxel (1 μM) and NM (160 μg/ml)	(16)
Hepatocellular carcinoma	TNF-a	Huh-7/Hep3B cells	p65, p50, and phosphorylated IκBα; Cleaved caspase-8/caspase-3/PARP;	Inhibition of cell proliferation Induction of apoptosis and G1 arrest	TNF-α (10 ng/mL) and NM (40 μg/ml)	(17)
Gallbladder cancer	Gemcitabine	NOZ cells	p65 and phosphorylated IκBα; Cleaved caspase-8/PARP;	Inhibition of cell proliferation Induction of apoptosis	Gemcitabine (1 μM) and NM (20 μg/ml)	(25)

**Table 2:** The anti-tumor activities of combination of NM and chemotherapy/radiotherapy *in vivo*.

**Tumor**	**Combined therapeutics**	**Administration**	**Animals**	**Targeting signaling factors**	**Biological functions**	**Drug concentration/dosage of IR**	**References**
Pancreatic cancer	Oxaliplatin	Injection	Xenografts nude mice	p65 and phosphorylated IκBα; Cleaved caspase-8/PARP; c-IAP1/2	Inhibition tumor growth Induction of apoptosis	Oxaliplatin (10 mg/kg) and NM (30 mg/kg)	(18)
	Gemcitabine	Injection	Xenografts nude mice	p65	Inhibition of tumor growth Induction of apoptosis	Gemcitabine (100 mg/kg) and NM (30 mg/kg)	(19)
	Gemcitabine and TNF-α	Injection	Xenografts nude mice	p50, p65, and phosphorylated IκBα; TNF-α-TNFR1; Cleaved caspase-8/caspase-3/PARP; c-IAP1/2;	Inhibition of tumor growth Induction of apoptosis	AxCAhTNF-α (1^*^10^8^ particle units in 20 μl of PBS) Gemcitabine (100 mg/kg) and NM (30 mg/kg)	(20)
	Gemcitabine (GEM) plus nab-paclitaxel (nPTX)	Injection	Xenografts BABL/c nude mice	p65; Cleaved caspase-8/caspase-3;	Inhibition of tumor growth Induction of apoptosis	GEM (50 mg/kg), nPTX (0.5 mg/kg) and NM (30 mg/kg)	(21)
	Paclitaxel	Injection	Xenografts nude mice	p65 and phosphorylated IκBα; Cleaved caspase-8/caspase-3;	Inhibition of tumor growth, and neovascularization Induction of apoptosis, Improvement of survival rate	Paclitaxel (20 mg/kg) and NM (30 mg/kg)	(22)
	IR	Injection	Xenografts nude mice	p65; Cleaved caspase-8/caspase-3; Mdm2/p53/ p21^Waf1/Cip1^;	Inhibition of tumor growth Induction of apoptosis	6 Gy and NM (30 mg/kg)	(23)
Colorectal cancer	Oxaliplatin	Injection	Xenografts nude mice	–	Inhibition of tumor growth Induction of apoptosis	Oxaliplatin (10 mg/kg) and NM (30 mg/kg)	(15)
	IR	Injection	Xenografts nude mice	p65	Inhibition of tumor growth Induction of apoptosis	5 Gy and NM (30 mg/kg)	(24)
Gastric cancer	Paclitaxel	Injection	Xenografts nude mice	p65 and phosphorylated IκBα; Cleaved caspase-8/caspase-3/ PARP;	Inhibition of tumor growth Induction of apoptosis Improvement of survival rate	Paclitaxel (20 mg/kg) and NM (30 mg/kg)	(16)
Hepatocellular carcinoma	TNF-α	Injection	Xenografts nude mice	p65 and phosphorylated IκBα; Cleaved caspase-8/caspase-3/PARP;	Inhibition of tumor growth Induction of apoptosis	AxCAhTNF-α (1^*^10^8^ particle units in 20 μl of PBS), and NM (30 mg/kg)	(17)
Gallbladder cancer	Gemcitabine	Injection	Xenografts nude mice	p65 and phosphorylated IκBα	Inhibition of tumor growth Induction of apoptosis	Gemcitabine (100 mg/kg) and NM (30 mg/kg)	(25)

Besides these classical strategies, some novel molecular inhibiters were confirmed to improve the antitumor effects of NM through pathways related to NF-κB signaling. Haruki et al. found that addition of glycogen synthase kinase-3 (GSK-3) inhibitor to NM significantly decreased the NF-κB/p65 activation and inhibited the cell proliferation in pancreatic cancers, though using GSK-3 inhibitor alone did not alleviate NF-κB activation. They also demonstrated that GSK-3β, one of the GSK-3 isomers dephosphorylated by protein phosphatase 2 (PPA2), was phosphorylated and acted as an inactive form after treating by NM. In that case, they further confirmed that NM combined with PP2A inhibitor significantly up-regulated phosphorylation of GSK-3β and promoted cellular apoptosis comparing to NM or PP2A alone, suggesting NM may improve the therapeutic outcomes of pancreatic cancer (38). Taken together, NM, alone or in combination with other anticancer treatments, plays a powerful role as a candidate intervention in NF-κB signaling for cancer therapy.

### TNFR1 Signaling

Apoptosis is a cellular suicide program that plays a pivotal role in the repression of cancer development. Among various mechanisms that contribute to apoptosis, the tumor necrosis factor receptor-1 (TNFR1) signaling pathway has emerged as one of the key mediators. TNFR1 is ubiquitously expressed on almost all cells of the human body and is the primary mediator stimulated by soluble TNF. When TNF binds and activates TNFR1, both pro-apoptosis pathways and anti-apoptosis pathways are affected, such as canonical NF-κB signaling based on subsequent formation of complexes. On the one hand, a pro-apoptotic cascade reaction consisting of multiple proteins, such as Fas-associated death domain (FADD), TNF receptor-associated death domain (TRADD), and the receptor-interacting protein kinase 1 (RIP1), occurs and forms caspase-8. All of these proteins are designated as complex II and further generate caspase-3 and polyadenosine ribose polymerase (PARP) to induce apoptosis (39, 40). Nevertheless, this cascade can be curbed by several factors, such as cellular inhibitor of apoptosis 1 and 2 (c-IAP1/2) (41), an NF-κB–regulated gene for suppressing apoptosis and promoting cell survival. On the other hand, the TNF-TNFR1 complex also recruits a series of proteins, including TNF receptor associated factor 2 (TRAF2), TRADD, c-IAP1/2, and RIP1. Among them, c-IAP1/2 is recruited by TRAF2 and further modifies RIP1 with polyubiquitin chains. This complex, known as complex I, leads to the activation of canonical NF-κB signaling pathways (39, 40). Therefore, targeting TNFR1-induced pro-apoptotic pathways to counteract the role of canonical NF-κB signaling pathways in stimulation of antiapoptotic transcriptional programs may represent a pivotal approach for cancer therapy.

The NM-induced apoptosis was identified in cultured cell and animal models, and demonstrated in human cancers such as pancreatic, colorectal, gastric, hepatocellular, and gallbladder cancers (12–18, 20–25). Multiple sources of data support that NM accelerates apoptosis in dual ways that not only prevent activation of NF-κB-regulated antiapoptotic process but also act on initiation of signaling molecules in TNFR1-induced apoptosis. Uwagawa et al. demonstrated that NM upregulates expression of TNFR1 in a dose-dependent manner and elevates phosphorylation of FADD on Ser-194 in a time-dependent manner. Based on activation of these two mediators and inhibition of NF-κB activation, expression of caspase-8 was upregulated to facilitate apoptosis (12). Furthermore, numerous studies have reported that NM plus chemotherapeutic agents or IR triggers expression of cleaved caspase-8, caspase-3, caspase-9, and PARP compared to using chemotherapy or IR alone in pancreatic, colorectal, gastric, gallbladder, and hepatocellular cancer, closely followed by downregulation of various forms of NF-κB (15, 16, 18, 20–22, 24, 25). Specifically, NM inhibits oxaliplatin-induced and TNF-α- and gemcitabine-induce c-IAP1/2 activity in pancreatic cancer (18, 20). These findings suggest that NM potentiates antitumor effects via inhibition of c-IAP1/2 expression in a positive feedback loop where restraining NF-κB activation negatively regulates c-IAP1/2 expression, which in turn represses activation of NF-κB and promotes apoptosis. Thus, an important consideration in anticancer fields is induction of downstream apoptotic pathways by NM.

### Inhibitory Effects Against Tryptase

Tryptase, a trypsin-like serine-proteinases with a molecular weight of 134 kDa, is the most abundant secretion of mast cells (MCs) (42). Tryptase contains a hydrolyzing peptide that binds to the carboxyl terminus of basic residues and a tetrameric structure consisting of non-covalently linked subunits, with an adequately active form stored in MCs (43). Two types of tryptase are expressed on human MCs, alpha and beta. Tryptase-α is the major circulating isoform, and tryptase-β is the major form stored in secretory granules (42). Normally, tryptase acts as an indicator to provide information about the distribution and activation status of MCs, so levels of serum tryptase may reflect disease states, such as allergy reaction, mastocytosis, and other inflammatory reactions (44, 45). Nevertheless, there is increasing evidence to support the view that tryptase is a vital mediator in biological pathways, including tissue remodeling and carcinogenesis (46).

Currently, an enormous number of studies have uncovered the association between MC-released tryptase and tumor progression, especially for neovascularization. For instance, a positive linear correlation was demonstrated between serum mast cell densities positive for tryptase (MCDPT) and microvascular density and endothelial area in patients undergoing surgery in colorectal, breast and gastric cancers, indicating that serum tryptase levels may represent a novel surrogate angiogenic marker in cancer patients (27, 47, 48). Involvement of tryptase in the degradation of extracellular matrix and the release of angiogenic factors, such as VEGF receptor and fibroblast growth factor-2, has been clearly demonstrated (49). Based on these findings, tryptase itself or the process that tryptase acts on might become potential drug targets for cancer therapy.

The pharmacological property of NM makes this compound an interesting candidate for new anticancer agent by inhibiting tryptase in colon and pancreatic cancer. Yoshii et al. identified that in colon carcinoma, NM blocks cell proliferation induced by treatment of tryptase or protease-activated receptor-2 (PAR-2) in a concentration-dependent fashion (27). PAR-2 is stimulated by tryptase (47) and mediates proliferative effects via phosphorylation of mitogen-activated protein kinase in colon carcinoma (27). In addition, PAR-2 upregulates MMP expression and plasminogen activator, leading to degradation of the extracellular matrix (48) and has become a hallmark for poor prognosis in combination with MCDPT. It is unclear whether NM directly inhibits expression of PAR-2 to exert its antiproliferative effects. Similar inhibitory effects of NM on tryptase-induced cell proliferation were observed in pancreatic cancer by Guo et al. They also demonstrated that NM possessed the ability to reverse tube formation caused by tryptase via inhibiting the expression of angiogenesis-related genes, angiopoietin-1 (ANGPT1) and TIE2 (26). It is well-known that ANGPT1 targets the TIE2 receptor as an agonist to regulate vascular maturation and stabilization (50). To date, investigations of NM inhibitory effects against tryptase for anti-cancer therapy are relatively scarce and limited to *in vitro* studies. Although application of NM is mentioned as a possible research direction, studies on the mechanism of targeting tryptase cancer therapy are in their infancy. More studies are needed both *in vitro* and *in vivo* in the future.

## Safety and Efficacy

NM is a synthetic low-molecular-weight serine protease inhibitor clinically used for acute pancreatitis and applied as an anticoagulant during extracorporeal circulation supportive treatment with a short half-life time (~23 min) (51). Evidence illustrating its clinical use for pancreatitis is suggested in animal models, illustrating that NM decreases the mortality of rats in an experimental acute pancreatitis model in a dose-dependent manner (Infusion at doses of 0.5–50 mg/kg/min) (6). In aspects of anticoagulation, a retrospective study indicated that NM could substitute for heparin in application of extracorporeal membrane oxygenation (ECMO), a novel rescue measure for circulatory and/or respiratory failure. Results showed that NM reduces anticoagulation values of patient to safe levels with an infused rate of 0.2–0.5 mg/kg per hour (52). In patients with acute kidney injury who received continuous renal replacement (CRRT) therapy, a single-center randomized study was performed and found that mean filter lifespan, a representation of treatment efficacy, was significantly longer in patients receiving NM than in those without NM, and no adverse events associated with NM administration were noted (53). For hematological malignancies patients diagnosed as DIC, the DIC resolution rates are 40.3 and 56.3% on days 7 and 14 after treatment of NM (0.06–0.20 mg/kg/day) (7). While compared to protease inhibitor gabexate mesylate (GM), there was no significant difference of DIC resolution rates between these two inhibitors. With respect to cancer therapy, three studies have been conducted to explore the possibility of NM's clinical application in pancreatic tumors treated with a combination of NM and gemcitabine chemotherapy. A phase I study was performed with 12 patients enrolled, and the regimen contained administration of gemcitabine at a fixed dose of 1,000 mg/m^2^ for 30 min on days 1, 8, and 15 of each 28-day cycle, accompanied by administration of NM starting via a port-catheter system for 24 h before infusion of gemcitabine. The starting dose of NM was 2.4 mg/kg with increments of 1.2 mg/kg until 4.8 mg/kg, and no patients experienced dose-limiting toxic effects at any level of incremental dose. In that case, the recommended dose of NM in combination with full-dose gemcitabine is 4.8 mg/kg (54). Next, based on the recommended dosage in a phase I trial, a single-arm phase II study occurred in a single center to evaluate treatment efficacy of 35 patients with unresectable and metastatic pancreatic cancer. The overall response rate was 17.1%, and 25% of patients who required opioids for cancer-related pain decreased their intake. From this evidence, the regimen revealed an effective improvement compared to standard chemotherapy with gemcitabine (55). Finally, in a retrospective single-center study, jaundice, ascites, high lymphocyte count, and high serum CA19-9 levels were investigated as poor prognostic factors for overall survival in patients with unresectable pancreatic cancer (56).

As an anticoagulant, the risk of bleeding is one of the most common adverse effects (>5%) associated with NM use. A retrospective study found that NM (500 mg was mixed with 5% dextrose and 250 mL water at an infusion rate of 20 mg/h) significantly increased bleeding complications (16.4%) in patients receiving ECMO compared to patients receiving heparin therapy (7.1%) (57). However, in 101 patients who received CRRT, use of NM tended to be correlated with decreased incidence of bleeding complications compared to the use of unfractionated heparin (6.6 vs. 16%) (58). Furthermore, headache (2.2%), nausea (1.3%), and fever (0.9%) were observed in a prospective, observational study including 832 patients with leukocytapheresis (59). Kim et al. reported a case of rare adverse reaction, anaphylactic shock, caused by NM in Korea after a 10-min infusion of NM for hemodialysis (60). In hematological malignancies patients with DIC, a retrospective study found that adverse events, such as hyperkalemia and hyponatremia, were more commonly observed in patients receiving NM (0.06–0.20 mg/kg/day) compared to GM (7). These findings indicate hints for exploration of adverse effects in cancer therapy. In unresectable pancreatic cancer, *in vivo* studies found that known adverse effects of NM, such as hyperkalemia, hyponatremia, and hepatopathy, were comparable between mice treated with NM at a dosage of 30 μg/g and PBS three times a week for 6 weeks (14). Moreover, another *in vivo* study of pancreatic cancer showed no significant difference in liver toxicity between combined injection of TNF-α and NM compared to injection of each compound alone (17). In a single-arm, single center, phase II trial, leukopenia and neutropenia primarily appeared in pancreatic cancer patients who received NM (4.8 mg/kg continuous regional arterial infusion) with gemcitabine (1,000 mg/m^2^ intravenously) on days 1, 8, and 15 (55). Thus, the superiority of NM in reducing adverse reactions is obvious in current clinical therapy, but randomized controlled studies are still necessary to detect adverse reactions and their rates of occurrence. In light of the above detailed findings, NM exhibits low toxicity and advantages based on combination therapy with traditional chemotherapeutic agents, suggesting that combined treatment of NM and chemotherapy may represent a novel promising strategy for cancer.

## Discussion

### The Potential Immune Therapy of NM

To date, there are several contractional points regarding immunomodulatory functions of NM. By reducing of granzyme activity and cytotoxic T lymphocytes cytolysis, NM suppressed local C5a/C3a production and attenuated T cell auto-reactivity in experimental autoimmune encephalomyelitis (61, 62). In stroke rat models, NM decreased the generation of proinflammatory factors TNF-α, interleukin-1β (IL-1β), inducible nitric oxide synthase and cyclo-oxygenase-2, and elicited the expression of anti-inflammatory mediators CD206, TGF-β, IL-10, and IL-4 (63). On the contrary, it was identified that NM play a role in immune activity enhancement. NM effectively increased the activity of T lymphocytes and natural killer cells in hepatic resection patients (64). Also, NM induced CD8 T-cell proliferation, and played a role as a co-adjuvant for peptide vaccination (65). As mentioned above, the major anti-cancer mechanism of NM was the down-regulation of TNF-α-induced NF-κB activation. Fujiwara et al. have demonstrated that NM could act as an immunotherapy sensitizer combined with TNF-α and gemcitabine in hepatocellular carcinoma and pancreatic cancer (17, 20, 21). Also, NM were demonstrated affected anti-angiogenic activity by MCs-derived tryptase in colon carcinoma (26, 27). In all, the existing evidence suggests that NM is inclined to show immune activation activity in antitumor therapy.

**Figure 2:**
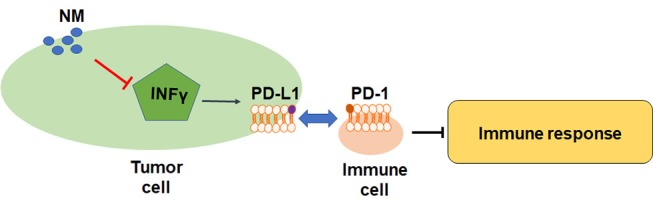
Mechanisms and biological functions of NM in immune response.

Although the role of NM in inflammatory-related pathway has been reported, the direct evidence of NM antitumor effects in immune cells is still not clarified. PD-L1 is a crucial factor in inhibition of T cell-mediated responses. The previous study has uncovered that NM reversed IFN-γ-induced up-regulation of PD-L1 in lung cancer and pancreatic cancer cells (Figure 2) (66). The pivotal immune escape factor human leukocyte antigen-ABC (HLA-ABC) may help differentiate benign from malignant indeterminate pulmonary lesions (67). The study of Homma et al. was confirmed that NM influenced IFN-γ-induced HLA-ABC up-regulation in lung cancer and pancreatic cancer (11), suggesting that NM may improve the treatment of immune resistant cancer. The inhibited effects of NM are associated with PD-L1-mediated immune evasion pathways including PAR-1, STAT1, STAT3, protein kinase B (Akt), interferon regulatory factors-1, extracellular signal-regulated kinase signals, but not NF-κB pathway. More interesting, inhibition of IFN-γ-induced PD-L1 upregulation was not found in above researches (68–72). Hence, the underlying mechanisms of how NM exerted its inhibition remains to be explored. More studies are warranted to figure out the correlation between NM and inflammatory factors and investigate the inhibition effects of NM in human tumors.

### A Brief Comparison Between NM and Other Protease Inhibitors

To further evaluate availability of NM for cancer therapy, herein, we briefly compared the function and clinical values between NM and other protease inhibitors. A series of synthetic serine protease inhibitors such as GM and bortezomib have been used for clinical application. Up to now, GM has been therapeutically used for DIC and acute pancreatitis in Asian countries (73). The antitumor effects of NM have been analyzed (74–76) and compared to GM in post-endoscopic retrograde cholangiopancreatography pancreatitis (PEP), wound healing and DIC (7, 77, 78). Similar to NM, GM exerted significant antitumorigenic effects on suppression of TNF-α-induced NF-κB expression and promotion of caspase-3, 7 activity in human pancreatic cancer cells (74). In addition, *in vitro* and *in vivo* studies reported that GM attenuated MMP activities and reduced cell growth, invasion and angiogenesis in colon cancer (75). These effects are also identified in a series of colorectal cancer cells with KRAS, BRAF and PIK3CA mutations (76). Since these genetic mutations have been emerged as predictive biomarkers for patients who might fail to EGFR-targeted therapy (79), these preliminary findings indicate that GM may represent a promising therapeutic agent for metastatic colorectal cancer. However, pharmacological mechanisms and clinical studies of GM on cancer therapy are still limited. Furthermore, the *in vitro* experiment demonstrated that NM could more effectively suppress the pancreatic protease activities than GM up to 10–100 times. A meta-analysis demonstrated the decreased risk of PEP was correlated with NM, while not associated with GM (77). The wound healing ability of NM was also identified to be more efficient than GM in rat models (78). However, the association between GM and NM were not observed in hematological malignancies-induced DIC. There were no significant differences in the DIC resolution rates between GM and NM groups, but the frequency of adverse effects was relatively higher in NM groups (7). Based on these discoveries, the comparable effects of NM and GM suggested the more potential usage of NM on cancer treatment, and further studies are still needed to validate safety and efficacy of NM in clinical trials.

The protease inhibitor bortezomib is the first drug approved by FDA for multiple myeloma treatment. Compared to bortezomib, NM showed the analogous inhibitory effects on NF-κB activation and IFN-γ-induced PD-L1 expression (66). Moreover, the preclinical and clinical data identified that NM could sensitize solid tumors to chemotherapy or classical chemo-radiotherapy (15–24, 37, 80). Nevertheless, FDA-approved proteases inhibitors such as bortezomib were observed mainly distributing in blood and/or bone marrow instead of solid tumors (3). Considering the limitations of current protease inhibitors, NM may apply for the treatment of solid tumor as a new aspect. In summary, these evidences indicate that prospects of NM for boarder clinical application are desirable even if currently only used in Asian countries.

### The Potential Clinical Uses of NM

So far, NM has been approved for treating diseases of digestive and hematological systems such as pancreatitis and DIC, and studies have confirmed potentiality clinical use of NM in various diseases. Herein, we summarized potential pharmacological action of NM in non-tumor diseases distributed in nervous, circulatory, respiratory system, and infectious. In *in vivo* models of nervous system, for example cerebral ischemia, transient middle cerebral artery occlusion rats indicated the blood-brain barrier (BBB) protective function of NM through inhibition of thrombin. The symptom such as neuronal damage, brain infarcts, brain oedema, and motor dysfunction caused by impaired BBB could be reduced after NM treatment (81, 82). Moreover, NM regulated cardiovascular functions through increasing nitric oxide generation via the Akt/eNOS signaling pathway, which indicating that NM might serve as a safeguard for preventing cardiovascular diseases (83). Asthma is one of the serious respiratory diseases all over the world. In rat models with asthma, the treatment of NM showed a decreased eosinophil and neutrophil infiltration, and decreased levels of inflammatory factors such as IL-5, IL-6, IL-13, and IL-17 in bronchoalveolar lavage fluid. Also, the nuclear NF-κB activity reduced in lung tissues (84). It indicated the applicable function of NM in respiratory systems. As for antimicrobial activity of NM, Inman et al. found that NM exerts a dose-dependent inhibition of chlamydial infection. The *in vitro* and *in vivo* models showed that NM could effectively minimized pathological features of chlamydial-induced arthritis, including inflammatory infiltration and joint damage (85). Additionally, NM could inhibit middle east respiratory syndrome coronavirus infections (86) and the ebola virus disease in *in vitro* studies (87). Obviously, it can be concluded that the diverse anti-inflammatory and NF-κB modulating abilities of NM may provide hopeful clinical applications for future pharmaceutical development.

### The Limitations of Research Progress

Currently, a detailed mechanism for how NM fosters inhibition of IκBα phosphorylation in malignant cancer cells remains elusive. Which molecules are regulated by NM in tryptase-mediated angiogenesis also remains unknown. At this point, the modulatory effects of NM on tumor growth are distinct, particularly when combined with chemo- and radiotherapy. Additional clinical studies are essential to identify the pathological significance of NM in cancer onset and to compare therapeutic responses between conventional therapy alone and in combination with NM. Furthermore, the immune involvements of NM are worthy to deeply dig either in immunoadjuvant therapy or directly in immunocyte sensitization.

## Conclusions

NM is a serine protease inhibitor associated with inhibition of tumor progression in various tumors. Herein, we summarized the pharmacological mechanisms and evaluated the clinical application of NM for cancer therapy. Current research demonstrates that NM blocks canonical NF-κB signaling, targets TNFR1-stimulated cleavage of caspase families, and the tryptase of mast cells to improve therapeutic outcome. As a potential novel anti-cancer agent, NM has been validated to ameliorate cancer therapy resistance and avoid immune resistance. Existing clinical data showed relatively preferable efficacy and safety in NM treatment. Compared with other proteases inhibitors, NM has comparable antitumor activities and advantages in human pancreatic and colorectal cancer. All above evidence highlight the superiority of NM. Nevertheless, the question remains whether NM could be used in treatment for cancer patients, and additional preclinical and clinical studies are essential to be further conducted.

## Author Contributions

YY, XC and ZX wrote this review article. XC, ZX, SZ, XW, WL, LQ, JW, XY, QS and ZG performed administrative and technical support. YY designed the study and contributed to manuscript preparation. All authors reviewed and approved the final version of the manuscript.

### Conflict of Interest Statement

The authors declare that the research was conducted in the absence of any commercial or financial relationships that could be construed as a potential conflict of interest.
